# Studying full-shift inhalation exposures to volatile organic compounds (VOCs) among Latino workers in very small-sized beauty salons and auto repair shops

**DOI:** 10.3389/fpubh.2023.1300677

**Published:** 2023-12-01

**Authors:** Nathan Lothrop, Flor Sandoval, Imelda Cortez, Rietta Wagoner, Nicolas Lopez-Galvez, Kimberly Parra, Ann Marie Wolf, Betsy C. Wertheim, Carolina Quijada, Amanda Lee, Stephanie Griffin, Melanie Bell, Scott Carvajal, Maia Ingram, Paloma Beamer

**Affiliations:** ^1^Mel and Enid Zuckerman College of Public Health, University of Arizona, Tucson, AZ, United States; ^2^Sonora Environmental Research Institute, Inc., Tucson, AZ, United States; ^3^University of Arizona Cancer Center, University of Arizona, Tucson, AZ, United States

**Keywords:** occupational health, community health workers, CBPR, exposure assessment, air pollution

## Abstract

**Background:**

One in every 200 US jobs is in a beauty salon or auto repair shop, where workers are regularly exposed to volatile organic compounds (VOCs) that may cause a range of short- and long-term health issues. In these shops, Latino workers are overrepresented and lack culturally and linguistically appropriate industrial hygiene resources. This leaves a gap in knowledge on inhalation exposures to VOCs in this hard-to-reach and ubiquitous worker population.

**Objective:**

Our goal was to recruit hard-to-reach, predominantly Spanish-speaking workers in beauty salons and auto repair shops and monitor total VOC inhalation exposures for over entire work shifts, with minimal impact on workers, clients, and business.

**Methods:**

We developed and refined measurement and exposure assessment methods for personal and area full-shift VOC inhalation exposures.

**Results:**

With minimal participant loss, we measured over 500 h of real-time, personal VOC exposures and recorded activities and other exposure factors for 47 participants, while also documenting chemical inventories and quantifying indoor area concentrations of specific VOCs among 10 auto repair shops and 10 beauty salons.

**Conclusion:**

Lessons learned from our study can assist future studies of inhalation exposures in other hard-to-reach occupational populations.

## 1 Introduction

Inhalation exposures to volatile organic compounds (VOCs) result in well-documented, often irreversible, health effects, including asthma (1, 2), cardiovascular disease (3), cancer (4, 5), adverse birth outcomes (6), and cognitive and neurological symptoms (7). Workers in beauty salons and auto repair shops are exposed to VOCs every day, yet they are almost completely unstudied in the United States (US) with some international studies (8, 9). Worker VOC exposures in *beauty* salons, which offer hair services in addition to other cosmetic work (e.g., nails), have only been studied using area monitors for select VOCs and post-shift urinalysis, which may be complicated by dermal exposure (10). In auto *repair* shops, which do mechanical repairs as well as auto body work, only a handful of specific VOCs in a small subset of work activities have been investigated in the US (11, 12). Meanwhile, one in every 200 US jobs is in a *beauty* salon or auto *repair* shop (13), and this does not include 264,600 self-employed beauty *salon* workers (0.2% of all jobs) (14). Further, *beauty* salons and auto *repair* shops employ over 150% more total workers (13) than the mutually exclusive, well-studied *nail* and auto *body* shops.

While VOC exposure risks are present in all beauty and auto repair shops, they are more acute in small businesses (<100 employees), which are less likely to hire industrial hygiene (IH) consultants (15, 16). Approximately 232,608 (>53%) beauty salon and 304,817 (77%) auto repair workers are employed in shops with <20 employees (17). Latino workers are over-represented in the small business workforce (18) in low-wage jobs with increased risk of occupational injury (19). Meanwhile, they are less likely to trust government agencies to ask for assistance (20), and there are few linguistically and culturally appropriate occupational health materials (21). Further, Latino beauty salon workers in the US use products and processes that are different from other ethnicities (22). Together, this has resulted in a critical gap in exposure knowledge about a sizeable portion of the US workforce.

Like previous studies with hard-to-reach occupational populations (20, 23, 24), our team utilized a collaborative community-academic partnership approach between the Sonora Environmental Research Institute, Inc. (SERI) and the University of Arizona (UA) to measure inhalation exposures to VOCs in predominantly Spanish-speaking workers in beauty salons and auto repair shops in southern metropolitan Tucson, Arizona. We developed methods to discretely measure personal total VOC exposures in real-time for the entire shift and document worker activities and other exposure factors and chemical inventories, all while minimizing impact on worker behaviors, business profitability, and client or customer comfort.

## 2 Materials and methods

### 2.1 Overview

SERI *promotoras* or community health workers recruited participants from beauty salons and auto repair shops, which were visited up to two times each. These bicultural and bilingual (Spanish and English) *promotoras* are largely from the community they work with, and have long-established trust and rapport with the small business community and with these specific trades (25). On the initial site visit, the *promotora* facilitated introductions of participants and UA staff. The *promotora* completed a site survey to assess relevant exposure factors, such as ventilation, and a chemical inventory of products at the business. During each visit, UA study personnel measured participants’ personal, real-time total VOC exposures for their whole shift, while recording exposure factors, including activity (e.g., bleaching hair, cleaning brakes) and any nearby activity, the room or location, and ventilation conditions. While inhalation personal protective measures were recorded, these were almost non-existent. To measure specific VOCs in the shop, air samples were collected at least once using an evacuated canister. The study took place from June through November 2018.

### 2.2 Recruitment

Study participants were recruited door-to-door at local businesses by SERI *promotoras* from the study area as described previously (26). *Promotoras* first obtained written permission from the business owner or manager to recruit at the shop. If an owner or manager was not present, the *promotoras* would revisit the shop when convenient. In each participating shop, the goal was to recruit owners, managers, or workers who expected to be at the shop most of the day, to monitor personal VOCs for four shifts per shop, which could include any combination of single or multiple measurements of each participant. This would be completed for 10 shops in each business sector (i.e., beauty and auto repair), for a total of 40 shifts per sector.

Participants had to be ≥18 years of age, able to speak and read Spanish or English, and expect to be employed at the shop for ≥3 months. The last requirement was instituted to ensure follow-up with each participant to have their sampling results reported back to them. Upon consenting, *promotoras* administered participant demographic and background surveys. Personnel completed all verbal and written communications in the participant’s language of choice (i.e., Spanish or English), and scheduled site visits for VOC monitoring at the business’ convenience. Study subjects were not compensated for their time but would receive their sampling results. All consent was obtained in writing. This study was approved by the University of Arizona Human Subjects Protection Program.

### 2.3 VOC monitoring site visits

#### 2.3.1 Measuring personal VOC exposures

Real-time monitoring of total VOC exposure was conducted using the ppbRAE 3,000 (RAE Systems, Inc., San Jose, CA), which can detect over 3,000 different VOCs with precision of one part per billion (ppb) with accuracy of 10 to 2,000 ppm: ±3% at calibration point. While the equipment representative suggested we bump test and calibrate monitors before each visit at the individual businesses, the study team was concerned this would add time to the site visit and present complicated liability issues, thereby reducing business recruitment and acceptance. Instead, in the hour before each site visit, UA staff bump tested and calibrated the ppbRAE monitors in a designated fume hood at the UA, as well as performing other diagnostic checks, all as per manufacturer instructions. Each monitor was ‘bumped’ or tested for accuracy with 100 parts per million (ppm) concentration of isobutylene. While possible to ‘translate’ a concentration in isobutylene to another VOC, it is impossible to know what chemical and what proportion during sampling. If a monitor failed the built-in accuracy criteria, it would lock until being successfully calibrated at 0, 100, and 1,000 ppm isobutylene concentrations. Each monitor’s pump was tested by blocking the flow of the running monitor, confirming it alarmed, and then restarting the device. A check of monitor lamp contamination was performed by cupping a hand around the probe without blocking the flow. If the concentration rose above 500 ppb and did not return to 0 ppb within 10 s, the monitor’s lamp was cleaned.

After we completed our first two sampling visits, we found the monitor’s internal clock drifted approximately 3 min every 24 h, making it difficult to link concentrations to recorded activities. To remedy this, we updated monitor time to Arizona Mountain Standard Time before every visit. To determine the monitor logging interval, we tested how a temporary source of VOCs (e.g., a burst of hair spray) would be logged at time intervals of 5, 10, 20, 40, and 60 s by all 7 study monitors. The 20 s logging interval was chosen as it both avoided increased ‘noise’ as found in smaller intervals, and still captured defined spikes, which were blunted or lost in larger intervals. All monitor alarm sounds were muted during sampling activities to increase comfort of participants and clients, and only visual cues were used.

**Figure 1:**
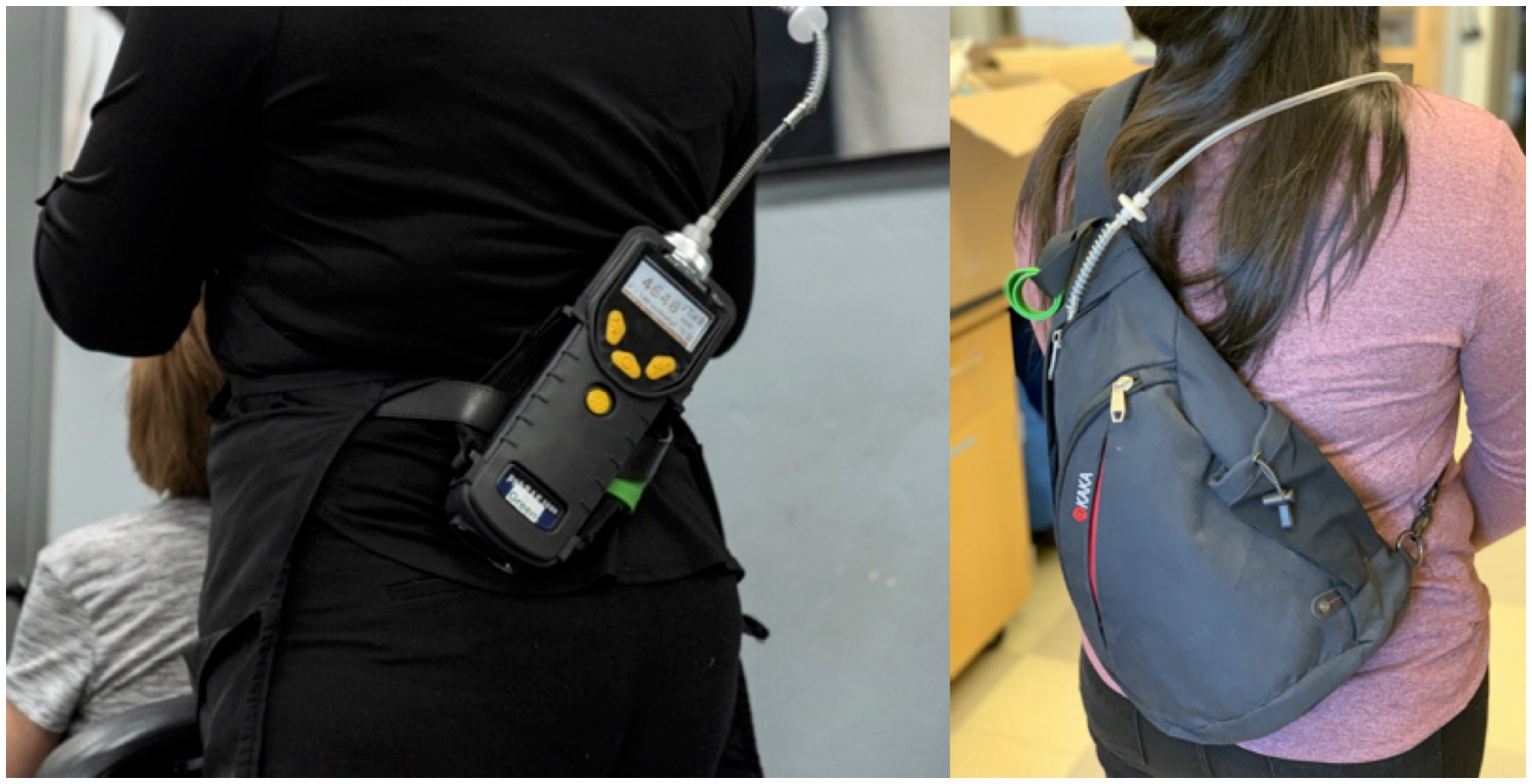
Participants wearing the ppbRAE monitor on a belt and in a sling backpack.

On the initial site visit, UA personnel would not enter the shop until the SERI *promotora* arrived to introduce them to the business owner and workers. To make the business and participants feel more comfortable, at least one of the same bilingual and bicultural field staff attended all site visits for each shop. After introductions, a ppbRAE was turned on for each participant and run outdoors for two minutes to obtain an ambient background concentration to correct for in later analysis. Study personnel fitted participants at their convenience with the monitor. Participants were given the option to wear the monitor either on a belt or in a sling backpack carried over one shoulder. Other wearable sampler setups (e.g., a leg holster, a 2-strap backpack) were developed and tested with the input of study team members with a range of education and work backgrounds, including those with relevant first- and secondhand work experience and friends, relatives, and acquaintances in these trades. Ultimately, it was decided that the belt and sling backpack were the most comfortable; most convenient to adjust; least esthetically objectionable for beauty salon workers; least likely to get caught on a moving part, a hazard for auto repair work; and most cost-effective to replace. To be easily identified by study staff, each monitor was color-coded with a matching silicone-coated snap-bracelet attached to the belt or pack (Figure 1). The participant could adjust how they wore the monitor as often they wanted, usually with the help of study staff.

The ppbRAE inlet was extended via tubing to collect air within the breathing zone of each participant (i.e., < 0.3 m radius of mouth and nose). The sampling train inlet was secured with an alligator clip to the lapel or apron to allow for movement without the train getting caught on nearby equipment, coworkers, or clients. As recommended by the monitor manufacturer, we used Versilon SE-200 fluorinated ethylene-propylene lined tubing (Saint-Gobain, Courbevoie, France) with two in-line filters with 0.3-micron pore sizes (one at the start and one at the end of the sampling train) to prevent debris or liquid from entering and subsequently damaging the monitor. All connections were done with twist-off, interlocking parts so that tubing length could be quickly changed without tools to reduce workflow disruption. While study staff pilot-tested sampling setups in both simulated and real-world scenarios, it was not discovered until after the first two sampling visits that tubing between the monitor probe tip and the first in-line filter would kink when participants would bend or kneel, which would result in a flow fault and loss of data. Subsequently, the section of tubing was fed through a 10 mm diameter spring to greatly reduce these incidents.

During sampling, study personnel viewed a handheld EchoView Host (RAE Systems, Inc., San Jose, CA), which showed the concentration and alarm status of all active ppbRAE monitors. To protect both participants and study personnel from excessive VOC exposures, we set a 15-min short-term exposure limit alarm of 1,000 ppm, as we were unaware of what VOCs we were measuring or their relative mixture. If a unit went into a short-term exposure limit alarm, all field staff were instructed to leave the building for fresh air and return when the alarm stopped. The owner or manager was made aware of this potential situation during recruitment. When the participant left the site property before the end of their shift (e.g., lunch break), they left the monitor running with field staff until returning. Concentrations recorded during this time off-site are redacted in later analysis (Moreno Ramírez et al., Unpublished). This was done to avoid having to recalibrate the monitor in the field. At the end of the shift, the monitor was turned off upon the participant removing it.

#### 2.3.2 Identifying key exposure factors

During each site visit, study staff observed and recorded participants’ activities and relevant information that could influence their VOC exposures for the duration of their shift. Based on the team’s experience in studying real-time, micro-level activities of children for exposure modeling (i.e., location, activity and intensity, surface) (27, 28), we developed paper activity logs to record exposure factors relevant to beauty and auto shops. Standardized menus of common activities (e.g., shampooing, changing oil), rooms where activities occurred, and ventilation conditions (e.g., ceiling fan, open window) were created based initially on SERI *promotora* experience and topical literature, and were updated with SERI and UA field experience (Supplementary Tables S1, S2). Staff mapped out rooms and ventilation setups or scenarios on paper at outset of the initial visit and used for all subsequent activity records at the site.

The activity log was set up such that participants could only complete a single activity in a single room at a time, but they could be affected by multiple ventilation conditions and nearby activities. A nearby activity was defined as any activity within 5 m of the participant, as based on how far a release of a pressurized spray released could be detected by a monitor. Changes in exposure factors were recorded down to the second. Whenever the participant changed activity, room, or ventilation scenario, or there was an activity occurring near the observed participant, a new line in the activity log would be created, with the start time marked. Later, the end time of activities would be inferred by the start time of the next. As possible, notes on product use and type were also taken (e.g., applying Brand X hair dye). Any changes in exposure factors, including quick changes between tasks common in beauty salons, lasting <10 s (e.g., sprayed brake cleaner for 3 s) were recorded in notes without beginning a new entry.

Study staff remained in the customer area or other location predesignated by the manager and did not follow participants when they left the area or were out of view. This was both to minimize participant workflow disruption, but also for field staff safety. When participants were out of view, no assumption could be made about their activity, room, or ventilation condition, and this was recorded as “out of view.” When the circumstances allowed, study staff inquired with participants about activities that were “out of view” to retroactively update these entries. The end of the participant’s work shift was recorded when the monitor was taken off and powered down. After the visit, hand-written activity logs were transcribed into a REDCap (REDCap, Vanderbilt University, Knoxville, TN) database by UA staff.

#### 2.3.3 Inventorying chemicals

To catalog chemicals in the shop during the first site visit for measuring exposures, a *promotora* photographed all products in use or in storage, as shown by the manager or workers. Images were captured and organized on an electronic tablet in a web-based REDCap form. Each image contained multiple products to improve efficiency by minimizing the number of photos. Study personnel also found containers were refilled with products or chemicals different than labeled or not labeled at all. To avoid potential contact with unknown chemical residues, study personnel did not touch product containers unless the shop manager gave explicit permission, and thus did not interrupt workflow. Additionally, study personnel were instructed not to enter any auto paint storage or use facility due to risk of acute diisocynate exposure, despite the risk of compromised image quality. After the conclusion of the first visit, study staff entered each product from the images into a database, along with transcribed chemical ingredient names, Chemical Abstracts Service (CAS) numbers, and amounts by volume from the product’s most recent Safety Data Sheet (SDS).

When the SDS was unavailable, ingredients were transcribed directly from matching images of the product label found on the vendor’s website. Ingredients were cataloged by CAS number. When a unique chemical had multiple CAS numbers, we chose the CAS number shared by CAS, PubChem, and the most recent exposure limit documents from the National Institute of Occupation Safety and Health and the American Conference of Government Industrial Hygienists. Additionally, ingredients with no CAS number, such as “Perfume” or “Proprietary” were also entered into this database. If a product was a variant or part of a line of items (e.g., different hair dye or auto oil viscosities) we documented these as a single product, unless it had different SDS chemical ingredients, in which case a distinct product was recorded for each distinct SDS.

#### 2.3.4 Measuring specific VOCs in shops

In order to quantify specific VOCs during site visits, we obtained time weighted average concentrations for 73 specific VOCs using a Summa canister and the US Environmental Protection Agency TO-15 analysis method (29). In each shop, a 6 L Summa canister (TestAmerica, Phoenix, AZ) was started at the beginning of the first participant’s shift. Summa canisters are evacuated vessels (~29.9 mm Hg) made of specially treated stainless steel that are designed to passively collect whole air samples once the valve is opened. The intake flow controller rate was selected so that 6 L of air would be sampled over the expected length of participant shifts, as communicated by the shop’s participants beforehand (typically 8–12 h), to avoid a scenario in which too little air was sampled, which would increase the risk of undetected VOC concentrations. After the desired collection time, the valve was closed, and the canister sent to a laboratory for analysis of the contents.

With input from the manager, in a conversation often facilitated by the *promotora*, we placed each Summa canister in the room with the most expected activity on the floor in a location that would not interrupt workflow. If allowed, additional Summa canister samplers were set up in areas that would remain closed for large parts of the day for specific activities, such as waxing rooms in beauty salons or paint booths in auto repair shops. When allowed, an additional Summa sample was collected during the second site visit to determine between-day variability. Locations of samplers were marked on the aforementioned site map. Canister sampling ended when the last worker wearing a monitor concluded their shift for the day. Duplicate samples (i.e., two adjacent canisters) were taken in 10% of shops. After sampling, Summa canisters were stored at room temperature until being transported via TestAmerica courier within 1 week of sampling to the TestAmerica facility in Phoenix, AZ.

## 3 Results

### 3.1 Recruitment and characteristics of study subjects

*Promotoras* enrolled 10 of 15 (67%) beauty and 11 of 23 (43%) auto shops they visited. One of the auto shops completed recruitment paperwork but left the study before sampling. In recruited beauty salons, 9 of 10 (90%) eligible owners and 14 of 25 (56%) workers consented to air sampling, while in auto shops, 6 of 10 (60%) eligible owners and 18 of 24 (75%) workers participated. Unlike in beauty salons, auto shop owners were eligible but seldom physically in the shop to participate in sampling. To ensure sufficient data quality and detail, two personal monitoring participants (one per field staff) were scheduled for each per day, necessitating two monitoring/sampling visits in all shops.

One auto shop requested only one sampling day, such that four workers were monitored at the same time by two field staff. In one auto shop, when the field staff arrived for the second visit, the owner said his shop was withdrawing from the study because his two participating workers had complained about workflow disruptions experienced on the first visit. When study staff asked the participants themselves what could be done differently, the owner answered for them, saying they were no longer in the study. The 24 auto repair participants were nearly all male, predominantly Latino, and 40 years old on average, while the 23 beauty salon participants were nearly all female (91%), all Latino, and slightly older (mean age 46.5 years) (Table 1). On average, auto repair work shifts were about an hour and a half shorter than beauty salons (5.8 vs. 7.2 h) as recorded during site visits.

**Table 1:** Characteristics of participants by shop type.

		Beauty salons (*N* = 10)	Auto repair shops (*N* = 10)
		*n* (%) or Mean ± SD	*n* (%) or Mean ± SD
Age (years)		46.5 *±* 9.31	40.0 *±* 13.7
Gender	Female	21 (91%)	0 (0%)
	Male	2 (9%)	23 (96%)
	Refused	0 (0%)	1 (4%)
Ethnicity	Latino	23 (100%)	18 (75%)
	Not Latino	0 (0%)	6 (25%)
Race	White	20 (87%)	21 (88%)
	Refused	3 (13%)	3 (12%)
Shop role	Owner/Manager	9 (39%)	6 (25%)
	Worker	14 (61%)	18 (75%)
Shift length (hours)		7.2 ± 1.5	5.8 ± 2.3

SD, standard deviation.

### 3.2 Measuring personal total VOC exposures

While wearing the VOC monitor, every participant changed from the belt to the backpack or vice versa at least once per shift. Salon participants seldom changed how it was worn (e.g., changing the shoulder for the backpack or shifting the monitor on the belt), yet auto shop workers did this often, especially lying down or contorting themselves to complete a repair. In these cases, field staff would help them reorient the monitor and the sampling train, including swapping out different lengths of tubing as needed to allow them to move freely. In addition, auto repair workers noted the backpack was hot to wear, which was not surprising given they do not work in climate-controlled spaces and sampling took place in summer and fall. Due to personal ergonomic issues, 1 of 24 auto repair and 2 of 23 beauty shop participants had to take off the backpack and hang it nearby for a portion of their shift. The only short-term exposure limit alarm occurred at an auto shop, but in this particular shop, the owner had barred study staff from entering the repair floor at any point. As a result, the participant was only alerted after they left the repair floor. Participant time weighted averages (TWAs) of total VOCs were higher in beauty salons (geometric mean = 2,035 ppb) compared to auto repair shops (832 ppb) (Supplementary Figure S1). In addition, the inter-quartile range of TWAs was smaller for auto repair subjects (2,884 ppb) versus beauty salon workers (4,116 ppb).

### 3.3 Identifying key exposure factors

In beauty salons, we recorded 277 total hours of activity data, while in auto repair shops, we logged 243 h (Table 2). Beauty participants changed activities, rooms, or ventilation conditions an average of 7.4 times/h, compared to 4.9 times/h for auto workers. In auto repair shops, we could not view or identify participant activity nearly 30% of the time, compared to just 11% in salons. When activity identification was possible, the most common activities in auto shops were mechanical repair (26% of time), administration (15%), and going on break (13%), while fluid services and cleaning (2%) were the least. In beauty salons, hair styling/cutting was the most frequent activity (37% of time), followed by going on break (18%) and hair processing (12%), while the least were cleaning (7%) and skin care (3%). While no nail technicians consented to air sampling, one was active in a salon for a portion of the visit. In auto shops, nearby activities occurred approximately 7% of the time, compared to 25% in beauty shops (i.e., predominantly hair styling/cutting and hair processing). Ventilation conditions varied widely; auto repair shops had 24 unique combinations, yet the most common scenarios involved an open door (40% of time) or local exhaust (35%) (Supplementary Table S3). Among salons, there were 19 distinct ventilation combinations, with participants working in a scenario with central HVAC 80% of the time (Supplementary Table S4).

**Table 2:** Activity definitions and durations in hours, ranked from most to least frequent, by shop type.

Auto repair shops		
Activity	General definition	Hours (%)
Unknown	Not observed or reliably deduced	70.0 (29%)
Mechanical repair	No active chemical use; no body work (e.g., rotate tires)	63.0 (26%)
Administration	Work aside from vehicle repair (e.g., talk on phone)	35.5 (15%)
Break	Not working (e.g., lunch)	31.6 (13%)
Painting, body or collision repair	Repairing or painting auto body	20.4 (8%)
Cleaning parts	Cleaning parts by any means (e.g., spray brake cleaner)	11.6 (5%)
Fluid services	Draining or replacing fluids (e.g., oil change)	5.62 (2%)
Cleaning	Cleaning shop itself	5.36 (2%)
All	--	243 (100%)

Beauty salons		
Activity	General definition	Hours (%)
Hair styling/cutting	Working on hair without any chemical	103 (37%)
Break	Not working (e.g., lunch)	51.2 (18%)
Hair processing	Working with any chemical on hair	32.6 (12%)
Administration	Work aside from beauty activities (e.g., talk on phone)	32.3 (12%)
Unknown	Not observed or reliably deduced	29.8 (11%)
Cleaning	Cleaning shop itself	20.2 (7%)
Skin care	Non-hair beauty work (e.g., waxing, nails)	7.74 (3%)
All	--	277 (100%)

### 3.4 Inventorying chemicals

We inventoried 304 total products or product variants from all businesses; of these, 293 were unique in brand, name, and variation (e.g., red vs. blue hair dye), with 114 (39%) in auto and 179 (61%) in beauty shops. Of these 293 unique products, 73 (25%) had no ingredient information, most of which were beauty products (*n* = 61), including 9 products imported from Mexico. Products with product naming and ingredient information comprised 536 chemicals, of which only 323 (60%) were uniquely identifiable with CAS numbers. We found that in both types of shops, specialty products used on a per-client or per-repair basis were not regularly in stock and obtained only 24–48 h prior to a scheduled appointment, which likely left many specialty products out of the chemical inventory. No shops had up-to-date chemical or product inventories available.

### 3.5 Measuring specific VOCs in shops

We utilized 16 Summa canisters in auto repair shops (13 in repair/overhaul areas and 3 in paint booths), with 3 shops measured on 2 different days, and 15 Summa canisters in beauty salons in the main work area, with 5 shops measured on 2 different days. No shops refused a sampler on the first visit, nor when the study team requested sampling additional locations or days. In auto shops, 31 unique chemicals were detected, and the most frequent was acetone (*n* = 16 samples), toluene (16), ethylbenzene (14), and xylene (14). Similarly, 31 unique chemicals were detected in beauty shops, and the most common were 2-propanol (*n* = 15 samples), acetone (13), and toluene (*n* = 11). Among detected chemicals, no concentrations were greater than relevant American Conference of Government Industrial Hygienists Threshold Limit Values (Supplementary Tables S5, S6).

## 4 Discussion

While other studies have investigated VOC exposures in US beauty salons via area monitoring (10) or personal sampling in auto repair shops for a small subset of activities (11, 12), we recruited a hard-to-reach population to study full-shift, inhalation exposures to VOCs in 20 very small auto repair shops and beauty salons among predominantly Spanish-speaking workers in southern metropolitan Tucson, AZ with the aid of *promotoras*. We measured real-time, personal, total VOC exposures and recorded second-by-second activities and other exposure factors (e.g., ventilation) for 47 participants, while also documenting chemical inventories and quantifying indoor area concentrations of specific VOCs. Innate differences between sectors and their work were borne out by participant demographics, work pace and duration, shop size and layout, and product regulations, which subsequently influenced how effectively we could identify personal exposure factors and the availability of product chemical information.

During recruitment, *promotoras* were more successful in enrolling beauty salons (67%) compared to auto shops (43%), yet participation rates were below that of previous work in similar shops (25). However, participation by workers within each business was higher in this study than other occupational health and exposure studies of Latino occupational populations (30, 31). This success speaks to the partnership with SERI *promotoras* and their ability to connect with participants as community liaisons, as shown in other settings (32). Further, no participants themselves explicitly dropped from the study, likely because of the relationships built by *promotoras* and the study’s discrete methods for assessing VOC exposures.

Acceptability of methods was demonstrated by no participants asking to leave the study, though some participants had to take off the monitor for a portion of their shift (3 of 47 total participants) to avoid aggravating previous injuries. Further, it was common to adjust the monitor setup, which would sometimes require study personnel help. Auto workers did this often, especially when changing positions to fit in or under a vehicle, while stylists did so but far less frequently. Given that our study is the first to complete real-time personal total VOC monitoring for entire work shifts in these US populations notably in auto repair, we found that our personal monitoring setups worked well but typically required study personnel to assist with adjustments. More pilot testing and input from those in the trades would benefit both the comfort of participants and data quality, while reducing staff time spent adjusting setups.

Generally, beauty shop workers changed tasks more often than mechanics, as evidenced by changes in activity notation per hour. It was common for stylists to move between multiple clients with different processes in multiple areas, while mechanics often focused on a single task for extended periods. One likely reason is the simultaneous presence of multiple customers in beauty shops, as compared to auto shops, which did not have a client actively waiting. Alternatively, compared to stylists in air-conditioned beauty shops, auto workers commonly work in temperatures of 38° C during this time of year (33) without climate control, potentially resulting in slower activity (34). In addition, moving quickly between rooms and tasks is more possible in small beauty shops, which were no more than 175 m^2^ in size, compared to auto shops which almost always encompassed multiple buildings, including one shop with its own salvage yard which had a total area of 5,240 m^2^.

As a result of layout differences, we had difficulty identifying activities in auto shops (29% were not observed), due to limited sight lines and that participants often worked in areas unsafe for study staff to enter (e.g., paint booth). The most extreme example was when one auto shop manager forbade field staff from entering the work area to watch participants minutes after the first site visit began. Field staff stayed in the customer waiting area which was in a different building, and would ask participants about their activities when they left the work area on breaks. Not surprisingly, the proportion of unknown activities ranged from 33–99% for participants in this shop. Limited identification of auto repair activities was also in part due to mechanics often working on the opposite side of, inside, or underneath a vehicle. As such, study staff may have felt less comfortable interrupting or talking with workers to ask about previous tasks. In comparison, only 11% of all activity in beauty shops was unknown.

While recording activities and exposure factors on paper forms offered the chance for noting precise detail that might be important, we found that transposing into REDCap was time consuming due to the transposer often needing to confirm detailed handwritten notes. Given the uncertainty of the breadth of tasks and products, the requisite detail and entry speed, and available application development expertise, creating a digital entry system was not feasible at the time. Future endeavors would benefit from creating a digital entry system as used previously to document similar levels of activity detail from recorded video (27, 28). While video recording would benefit data entry accuracy, it was not feasible for this vulnerable population. A SERI *promotora* with significant experience in working with such shops said it would cause owners, managers, and workers to more likely avoid the study because of privacy and liability concerns.

In creating chemical inventories by photographing products in the store and transposing them into a database later, we saved time during documentation at the site visit but expended much more staff effort in database creation. Unlike other studies, which found and used SDS records in shops to calculate exposures (35), we never found updated versions in auto shops (as required by local ordinance) nor in salons. While this may speak to the degree of local fire code enforcement (36) or a knowledge gap among some owners, it also makes it clear that relying on such records alone is not enough for future studies. While most SDSs for auto products listed identifiable chemicals, one in four beauty products either had no listed chemical ingredients or contained unidentifiable compounds, such as “Fragrance.”

Companies cannot be forced to disclose “trade secrets” under the Fair Packaging and Label Act if the chemical is deemed non-toxic and used solely in cosmetics (37). Unsurprisingly, all salons had at least one product with unidentifiable chemical ingredients, which carries an important lesson about the potential unknown exposures in the less-regulated arena of beauty salon products. Future research and public health will benefit from California’s Assembly Bill 2,775, which requires professional cosmetics sold in the state after July 1st, 2020 to list all chemical ingredients (38). This will likely affect products sold throughout the US, given California is 12% of the US by population (39), which should result in more informative ingredient lists.

Interestingly, while VOCs were the exposure of interest because of their known acute and chronic health effects, formaldehyde (a VOC not measured by any of our monitors) and hydrogen peroxide were also considered based on previous *promotora* experience in beauty salons and some enrolled salon participants asking about these chemicals. Ultimately, the team decided not to sample these chemicals given the reliability of available real-time instruments and other sampling methods in an already difficult situation, not to mention nuanced results interpretation and unbudgeted material and personnel cost. However, it was clear that these contaminants were of upmost concern for stylists and future work should consider sampling for them if the study allows.

In conclusion, we were able to study full-shift VOC exposures for 47 participants in a total of 20 very small auto repair shops and beauty salons in a predominantly Spanish-speaking population using discrete methods, resulting in almost no participant or shop dropout. While this study was completed prior to the COVID-19 pandemic, which inarguably has and will continue to alter work tasks and other exposure factors (e.g., cleaning practices, number of clients, ventilation) (40), methods developed in this study are no less pertinent. Lessons learned here can assist future studies of inhalation exposures in other hard-to-reach occupational populations.

## Data availability statement

The original contributions presented in the study are included in the article/Supplementary material, further inquiries can be directed to the corresponding author.

## Ethics statement

The studies involving humans were approved by University of Arizona Human Subjects Protection Program. The studies were conducted in accordance with the local legislation and institutional requirements. The participants provided their written informed consent to participate in this study.

## Author contributions

NL: Formal analysis, Investigation, Methodology, Project administration, Writing – original draft, Writing – review & editing. FS: Investigation, Methodology, Project administration, Writing – review & editing. IC: Investigation, Methodology, Project administration, Writing – review & editing. RW: Investigation, Methodology, Writing – review & editing. NL-G: Investigation, Methodology, Writing – review & editing, Validation. KP: Investigation, Methodology, Writing – review & editing. AW: Methodology, Writing – review & editing, Conceptualization, Funding acquisition, Project administration. BW: Methodology, Writing – review & editing, Data curation, Formal analysis, Software. CQ: Methodology, Writing – review & editing, Investigation. AL: Methodology, Writing – review & editing, Investigation. SG: Methodology, Writing – review & editing. MB: Conceptualization, Data curation, Formal analysis, Funding acquisition, Methodology, Project administration, Writing – review & editing. SC: Conceptualization, Funding acquisition, Methodology, Writing – review & editing. MI: Conceptualization, Funding acquisition, Methodology, Writing – review & editing. PB: Conceptualization, Formal analysis, Funding acquisition, Methodology, Project administration, Supervision, Writing – review & editing.
