# Supplementary material for: Studying full-shift inhalation exposures to volatile organic compounds (VOCs) among Latino workers in very small-sized beauty salons and auto repair shops
Source: Front Public Health. 2023 Dec 1;11:1300677. doi: 10.3389/fpubh.2023.1300677 (PMC10722412; doi:10.3389/fpubh.2023.1300677)
Supplement: Supplementary file 1 [file Table_1.DOCX]

Supplementary Material

Studying Full-shift Inhalation Exposures to Volatile Organic Compounds (VOCs) among Latino Workers in Very Small-sized Beauty Salons and Auto Repair Shops

Nathan Lothrop^1*^, Flor Sandoval^2^, Imelda Cortez^2^, Rietta Wagoner^1^, Nicolas Lopez-Galvez^1^, Kimberly Parra^1^, Ann Marie Wolf^2^, Betsy C. Wertheim^3^, Carolina Quijada^1^, Amanda Lee^1^, Stephanie Griffin^1^, Melanie Bell^1^, Scott Carvajal^1^, Maia Ingram^1^, Paloma Beamer1^1^

^1^Mel and Enid Zuckerman College of Public Health, University of Arizona, Tucson, Arizona, USA

^2^Sonora Environmental Research Institute, Inc., Tucson, Arizona, USA

^3^University of Arizona Cancer Center, University of Arizona, Tucson, Arizona, USA

*** Correspondence:**Nathan Lothrop
lothrop@arizona.edu

# Supplementary Data

Supplementary data is available for download.

# Supplementary Figures and Tables

**Table S1. Recorded Micro and Macro Activity Categories for Auto Repair Shops.**

| **Recorded Micro Activity** | **Macro Activity Category** |
| --- | --- |
| Body Repair | Painting, Body or Collision Repair |
| Painting/Paint Stripping | Painting, Body or Collision Repair |
| Oil Change | Fluid Services |
| Fluid Services (e.g., transmission, antifreeze, fuel) | Fluid Services |
| Degreasing/Cleaning | Cleaning Parts |
| Vapor Degreasing | Cleaning Parts |
| Brake Cleaning | Cleaning Parts |
| Disposal of Fuel/Fluids | Fluid Services |
| Dispensing Fuel | Fluid Services |
| Engine Repair | Mechanical Repair |
| Transmission Repair | Mechanical Repair |
| Tire Repair | Mechanical Repair |
| Windshield Repair | Mechanical Repair |
| Airbag Repair | Mechanical Repair |
| Clean Up/Housekeeping | Cleaning |
| Administration | Administration |
| Taking Break | Break |
| Unknown | Unknown |
| Out of View | Unknown |

**Table S2. Recorded Micro and Macro Activity Categories for Beauty Salons.**

| **Recorded Micro Activity** | **Macro Activity Category** |
| --- | --- |
| Hair-Dye/Strip/Color | Hair Processing |
| Hair-Perming/Relaxing | Hair Processing |
| Hair-Weave/Wigs/Extensions | Hair Styling/Cutting |
| Hair-Styling/Cutting | Hair Styling/Cutting |
| Hair-Brazilian Blowout | Hair Processing |
| Hair-Keratin Treatment | Hair Processing |
| Hair-Shampoo/Rinse | Hair Processing |
| Eyelash (e.g., extensions, lift/perm, tint/dye) | Skin Care |
| Body Waxing | Skin Care |
| Face-Microblading/Tattoo | Skin Care |
| Face-Makeup | Skin Care |
| Face-Chemical Peel | Skin Care |
| Nails-Acrylic (Note Application/Removal) | Nails |
| Nails-Gel/Teflon (Note Application/Removal) | Nails |
| Nails-Powder Dip (Note Application/Removal) | Nails |
| Nails-Polish (Note Application/Removal) | Nails |
| Nails-Nail Art (Note Application/Removal) | Nails |
| Nails-Unknown (Note Application/Removal) | Nails |
| Clean Up/Housekeeping | Cleaning |
| Administration | Administration |
| Taking Break | Break |
| Unknown | Unknown |
| Out of View | Unknown |

**Table S3. Duration of Unique Ventilation Condition Combinations in 10 Auto Repair Shops.**

| **Ventilation Scenario** | **Length (Hours)** | **Hours (%)** |
| --- | --- | --- |
| Ceiling fan + Open windows/doors | 0.83 | 0.34 |
| Ceiling fan only | 0.04 | 0.02 |
| Cent AC only | 3.53 | 1.45 |
| Floor fan + open window/door + ceiling fan | 0.07 | 0.03 |
| Floor fan + open window/door + outside | 0.03 | 0.01 |
| Floor fan + open window/door + paint booth | 1.71 | 0.70 |
| Floor fan + open window/door + swap cooler | 9.81 | 4.03 |
| Floor fan + open windows/doors | 14.3 | 5.86 |
| Floor fan + outside | 5.54 | 2.28 |
| Floor fan + outside + paint booth | 0.04 | 0.02 |
| Floor fan + outside + swap cooler | 0.03 | 0.01 |
| Floor fan + swap cooler | 0.25 | 0.10 |
| Floor fan + window/door AC + paint booth | 0.79 | 0.32 |
| Floor fan only | 3.09 | 1.27 |
| Local exhaust only | 86.7 | 35.7 |
| Open windows/doors + cent AC | 0.12 | 0.05 |
| Open windows/doors + outside | 7.25 | 2.98 |
| Open windows/doors + swap cooler | 29.7 | 12.2 |
| Open windows/doors only | 33.6 | 13.8 |
| Other | 27.1 | 11.2 |
| Outside + swap cooler | 0.63 | 0.26 |
| Paint booth only | 7.78 | 3.20 |
| Swamp only | 3.89 | 1.60 |
| Window AC | 6.28 | 2.59 |
| *Total* | *243* | *100* |

**Table S4. Duration of Unique Ventilation Condition Combinations in 10 Beauty Salons.**

| **Ventilation Scenario** | **Length (Hours)** | **Hours (%)** |
| --- | --- | --- |
| Ceiling fan + floor fan | 0.07 | 0.03 |
| Ceiling fan + open windows/doors | 1.58 | 0.57 |
| Ceiling fan only | 2.02 | 0.72 |
| Cent AC + ceiling fan | 33.2 | 11.9 |
| Cent AC + ceiling fan + floor fan | 21.4 | 7.66 |
| Cent AC + ceiling fan + open windows/doors | 1.58 | 0.56 |
| Cent AC + floor fan | 11.1 | 3.98 |
| Cent AC + floor fan + swap cooler | 0.02 | 0.01 |
| Cent AC only | 158 | 56.7 |
| Floor fan + open windows/doors | 10.7 | 3.84 |
| Floor fan + open windows/doors + window AC | 7.55 | 2.70 |
| Floor fan + swap cooler + open windows/doors | 0.12 | 0.04 |
| Floor fan + window AC | 0.06 | 0.02 |
| Floor fan only | 3.64 | 1.30 |
| Open windows/doors + cent AC | 0.15 | 0.05 |
| Other | 0.31 | 0.11 |
| Outside only | 3.46 | 1.24 |
| Swap cooler only | 0.04 | 0.02 |
| Window AC | 24.1 | 8.61 |
| *Total Duration* | *279* | *100* |

**Table S5. Characteristics of Chemicals Detected in Auto Repair Shops.**

| **Chemical** | **Median (ppb)** | **Range (ppb)** | **% Detected** | **ACGIH TLV (ppb)** |
| --- | --- | --- | --- | --- |
| 1,1,2-Trichloroethane | 88.0 | 88.0 - 88.0 | 6% | 10,000 |
| 1,2,4-Trimethylbenzene | 3.25 | 1.20 - 4.80 | 25% | 25,000 |
| 1,3-Butadiene | 5.90 | 5.90 - 5.90 | 6% | 2,000 |
| 1,3,5-Trimethylbenzene | 0.91 | 0.61 - 1.20 | 12% | 25,000 |
| 2-Butanone (MEK) | 1.04 | 0.96 - 150 | 25% | 200,000 |
| 2-Propanol | 15.0 | 2.50 - 22.0 | 31% | 200,000 |
| 2,2,4-Trimethylpentane | 1.55 | 0.56 - 3.50 | 37% | 300,000 |
| 4-Ethyltoluene | 0.99 | 0.68 - 2.20 | 25% | No TLV |
| 4-Methyl-2-pentanone (MIBK) | 30.5 | 6.30 - 82.0 | 25% | 20,000 |
| Acetone | 140 | 6.90 – 3,800 | 100% | 250,000 |
| Benzene | 1.30 | 0.58 - 7.90 | 50% | 500 |
| Chloroform | 3.60 | 3.60 - 3.60 | 6% | 10,000 |
| Chloromethane | 1.00 | 1.00 - 1.00 | 6% | 100,000 |
| Cyclohexane | 3.40 | 0.84 - 6.60 | 62% | 100,000 |
| Dichlorodifluoromethane | 1.61 | 0.42 - 2.80 | 12% | 1,000,000 |
| Ethyl acetate | 4.65 | 0.32 - 22.0 | 50% | 400,000 |
| Ethylbenzene | 3.10 | 0.77 – 150 | 87% | 20,000 |
| Cumene | 0.92 | 0.92 - 0.92 | 6% | 50,000 |
| m,p-Xylenes | 10.8 | 2.40 – 570 | 87% | 100,000 |
| n-Heptane | 3.70 | 1.10 - 14.0 | 37% | 400,000 |
| n-Hexane | 3.70 | 2.10 - 12.0 | 43% | 50,000 |
| n-Nonane | 120 | 2.00 – 140 | 18% | 200,000 |
| n-Octane | 4.95 | 0.41 – 210 | 75% | 300,000 |
| n-Propylbenzene | 0.81 | 0.81 - 0.81 | 6% | No TLV |
| o-Xylene | 4.00 | 0.83 – 150 | 87% | 100,000 |
| Propene | 3.10 | 0.60 - 23.0 | 43% | 500,000 |
| Styrene | 80.5 | 41.0 – 120 | 12% | 20,000 |
| Tetrachloroethene | 5.80 | 5.80 - 5.80 | 6% | 25,000 |
| Tetrahydrofuran | 9.30 | 2.10 – 140 | 18% | 50,000 |
| Toluene | 71.0 | 1.60 – 3,200 | 100% | 20,000 |
| trans-1,2-Dichloroethene | 24.6 | 6.30 - 43.0 | 12% | 200,000 |

**Table S6. Characteristics of Chemicals Detected in Beauty Salons.**

| **Chemical** | **Median (ppb)** | **Range (ppb)** | **% Detected** | **ACGIH TLV (ppb)** |
| --- | --- | --- | --- | --- |
| 1,2,4-Trimethylbenzene | 9.35 | 2.70 - 16.0 | 13% | 25,000 |
| 1,3,5-Trimethylbenzene | 3.80 | 1.40 - 6.20 | 13% | 25,000 |
| 1,4-Dichlorobenzene | 1.77 | 0.25 - 3.30 | 13% | 10,000 |
| 2-Butanone (MEK) | 1.60 | 0.43 - 19.0 | 53% | 200,000 |
| 2-Propanol | 55.0 | 5.20 – 3,600 | 100% | 200,000 |
| 2,2,4-Trimethylpentane | 17.0 | 17.0 - 17.0 | 6% | 300,000 |
| 4-Ethyltoluene | 4.25 | 1.20 - 7.30 | 13% | No TLV |
| Acetone | 83.0 | 6.00 – 3,800 | 86% | 250,000 |
| Benzene | 7.20 | 1.20 - 16.0 | 20% | 500 |
| Carbon disulfide | 0.59 | 0.49 - 1.10 | 20% | 1,000 |
| Chloroform | 0.74 | 0.27 - 1.20 | 13% | 10,000 |
| Cyclohexane | 14.0 | 1.50 - 22.0 | 20% | 100,000 |
| Dichlorodifluoromethane | 0.27 | 0.18 - 0.31 | 40% | 1,000,000 |
| Ethyl acetate | 8.00 | 0.39 - 60.0 | 66% | 400,000 |
| Ethylbenzene | 1.10 | 0.35 - 5.30 | 20% | 20,000 |
| Cumene | 0.89 | 0.89 - 0.89 | 6% | 50,000 |
| m,p-Xylenes | 3.20 | 0.50 - 22.0 | 20% | 100,000 |
| Methylene Chloride | 4.33 | 0.67 - 8.00 | 13% | 50,000 |
| n-Heptane | 36.0 | 36.0 - 36.0 | 6% | 400,000 |
| n-Hexane | 0.51 | 0.19 - 110 | 40% | 50,000 |
| n-Nonane | 0.99 | 0.13 - 2.20 | 20% | 200,000 |
| n-Octane | 2.89 | 0.18 - 5.60 | 13% | 300,000 |
| n-Propylbenzene | 4.50 | 4.50 - 4.50 | 6% | No TLV |
| Naphthalene | 0.20 | 0.20 - 0.20 | 6% | 10,000 |
| o-Xylene | 0.87 | 0.18 - 7.40 | 26% | 100,000 |
| Propene | 12.0 | 0.49 - 89.0 | 60% | 500,000 |
| Styrene | 1.59 | 0.98 - 2.20 | 13% | 20,000 |
| Tetrachloroethene | 0.81 | 0.81 - 0.81 | 6% | 25,000 |
| Tetrahydrofuran | 2.40 | 2.40 - 2.40 | 6% | 50,000 |
| Toluene | 0.99 | 0.14 - 160 | 73% | 20,000 |
| Trichlorofluoromethane | 0.13 | 0.11 - 0.23 | 33% | 1,000,000 |

## Supplementary Figures


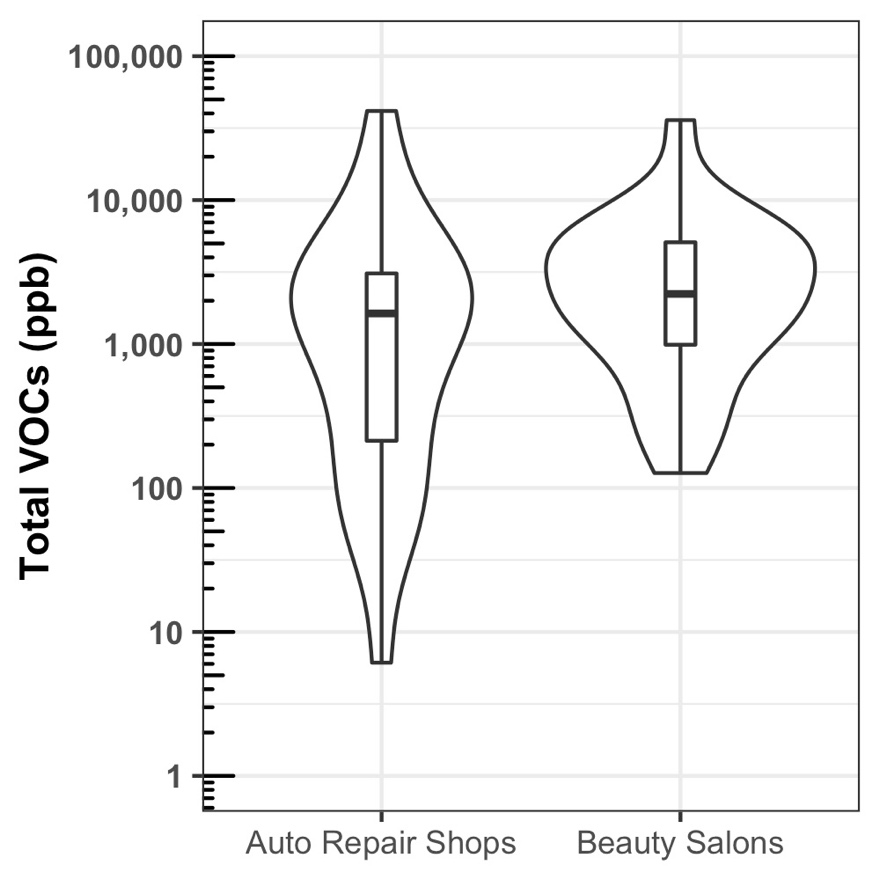


**Figure S1**. **Participant** **Time Weighted Average Exposures of Total VOCs.** Violin Plots Illustrate the Distribution of Data Points. In Box Plots, the Bold Line in the Center of the Box is the Median. The Lower and Upper Hinges Represent the 25th and 75th Percentiles. The Upper Whisker Extends from the Hinge to the Largest Value no further than 1.5 * Inter-quartile Range (IQR) of the Hinge. The Lower Whisker Extends from the Hinge to the Smallest Value at most 1.5 * IQR of the Hinge.
